# What if health care worked like everything else?

**DOI:** 10.1186/s13584-021-00503-8

**Published:** 2021-11-25

**Authors:** Shira Fischer

**Affiliations:** RAND Corporation, 20 Park Plaza, Suite 920, Boston, MA 02116 USA

## Abstract

Doctors use up-to-date communication technology in their personal lives, but the tools they use to communicate with professional colleagues are much more limited. In light of new research exploring the use of WhatsApp in Israel, this commentary explores the barriers to such use and the technological, legal, and cultural factors that enable adoption of such technologies for medical communication. Common tools can be used for professional collaboration but must be adapted for that use as well as culturally acceptable for broad implementation.

Over 10 years ago, a video—depicting what booking airline travel would look like if it were similar to health care in the United States—went viral. The 7-min clip, created by TheNewAltons [1], was based on a column written by Jonathan Rausch [2]. The parody successfully highlighted many of the weaknesses, challenges, and frustrations of the U.S. health care system. The caller, who wants to fly to Portland in October 23, is told that the next availability is in January, and he can only fly to Chicago, because a different company handles the West Coast leg of the trip, who he’ll have to call separately. The agent asks for his travel history, which he says he’s filled out many times. She offers to fax him a release form so he can contact the luggage carrier and release his travel information, since the airline doesn’t handle luggage (even in 2009, he mocks the use of faxes). She then asks for his travel insurance and then apologizes that her company doesn’t work with that insurance. When, frustrated, he begins to complain about the process, the agent explains how many airports and travelers and moving pieces there are. Calm and condescending, she tells him, “Getting everyone to coordinate services and exchange information just isn’t realistic in a business as complicated as travel.”

Well, that’s what they say in the U.S. (and many other countries) about health care, and unfortunately, not much has changed since then. There is some improvement in data sharing through some electronic health record (EHR) systems, and patient portals have improved patient access to records, especially with federal rules now mandating online access to most medical notes. Certain health care systems, including Israel’s four HMOs, have smartphone apps that make results retrieval and appointment booking very easy. However, sharing of patient data between health care systems remains a challenge, and in the United States, where many patients see providers in multiple systems, data is siloed and coordination is fragmented.

Of course, instead of seeing health care’s weaknesses applied to other fields, as amusing and illuminating as it may be, what we really want to see is the best of other fields applied to health care. Can we get the interoperability of ATMs, the personalization of websites like Amazon, and the accessibility of text messaging applied to health care? In 2021, you can order almost anything to your home over the Internet from your cell phone; you can text with your cell phone company; you can find services for your roof or your pet through an app. But your doctor still has to sit on hold with another doctor’s office in order to get professional advice.

In a recent IJHPR article, Edward Barayev and colleagues begin to describe what a different model could look like [3]. In Israel, WhatsApp is used more frequently than regular SMS messaging, with a 2020 report by Bezek, an Israeli telecommunications company, finding 91% of the country uses it [4]. (In fact, during the recent downtime of Facebook, Instagram, and WhatsApp, some people joked that they had forgotten that SMS messaging still existed). According to the results of Barayev and colleagues’ 2019 survey of 201 doctors (primary care doctors [PCPs] and specialists, 59% response rate), WhatsApp use was also very high among physicians, and not just for personal messaging: over 85% used WhatsApp at least once a day for professional use, while about the same percent reported low professional use of Facebook. PCPs preferred consulting specialists using WhatsApp over phone calls by almost 2 to 1.

Why is this medium preferred? The surveyed physicians reported a number of advantages to WhatsApp compared to other means of communication. WhatsApp allows for easy sharing of videos and images, such as in the case of rashes. It allows users to edit messages, so that they can think about the answer. It also allows primary care physicians and others to find the relevant information when a specialist asks something that they don’t know when they finally reach a specialist on the phone. And it allows physicians to respond when it is convenient instead of interrupting their other activities. The doctors also report that these remote, asynchronous consultations lead to fewer referrals, meaning that—at least anecdotally—this approach is also cost-saving.

So, what are the barriers to more physicians adopting this convenient approach? As the authors note, patient confidentiality and lack of documentation are the biggest concerns. While texting systems exist that are compliant with privacy laws, the commonly used ones, and therefore the most convenient ones, like SMS and WhatsApp, are not.

The Israel Defense Forces (IDF) provided guidance for WhatsApp use to protect identifiable information, but even so, each user needs to take care to protect privacy, particularly when sharing images or reports. Furthermore, any official documentation of the consult would have to be created manually after the conversation. However, this is less of a concern if the focus is on outcomes rather than billing (as in the IDF Medical Corps), and the same manual documentation is necessary following a phone consultation, so this is less of a barrier.

A third key feature is that the tool has to be commonly used. Texting, whether via SMS or WhatsApp, is ubiquitous, but there is no shared secure platform to which all physicians have access. In the US, decentralization means that doctors at one hospital may have an internal system for messaging each other that is secure and easily documented, such as through the Epic In Basket, but this system does not extend to all physicians with whom they may want to speak or whom their patients see. It is unlikely that they would have the direct phone number for any of those physicians and generally have to call and wait on hold to reach the relevant colleague. Thus the default is email or the office telephone.

On the other hand, specialists don’t want to be answering questions at all hours, and contacting them by WhatsApp may make them feel pressure to do so, even when not urgent. This is problematic, especially in light of the burnout that many providers are feeling. Also, in this Israeli military physician population, PCPs seemed to know how to contact specialists, or their numbers were available. That may not be the case in other systems.

Perhaps most important are the country-specific professional norms. A short message with a medical question containing no identifying information to a known colleague breaks no technical rules, but it is not something American doctors are used to doing. And, in a health care system that does not penalize them for making referrals, they have a low bar to send a patient for a specialist referral: there is no cost to them, it quickly moves the patient issue towards resolution, and it in fact takes less time than consulting a specialist themselves, even if it costs the system money and the patient time. Moreover, to text a consult, you need to know the specialist’s personal cell phone. Thus, texting between PCPs and specialists in the US is rare, with most such consults conducted via phone calls to a work number (and then a page) or via email to a work account.

However, there are other cultural patterns that seem more parallel with WhatsApp consultations. For example, the Physician’s Mom Group, or PMG, is a Facebook group of over 100,000 doctors. Like doctors WhatsApping other doctors, PMG members use up-to-date communication technology to expand access to expertise. An ED doctor in Arizona covering an overnight shift may post a picture of a bad wound (noting she has permission of the patient) or a confusing case with a complicated but deidentified medical history, and she will receive rapid expert responses from doctors all over the world [5]. Issues of privacy are handled by community standards and any documentation is left up to the physician. Unlike the direct messaging described by Bayarev et al., there is no way to know that a response from someone on PMG is authentic at all. Despite careful screening before anyone is admitted to the group, there is still no guarantee that the posters are whom they say they are, given the platform, or that the answer provided does come from a doctor in good standing. On the other hand, the platform gives access to thousands of doctors of all specialties at any hour of the day, way beyond the reach of WhatsApp to known doctors. And like the quick text message, it suggests to the questioning doctor what other diagnoses to consider, what additional tests to consider, and what might have been missed, after which they may still formally consult a specialist, but perhaps with more information when doing so.

Facebook physician groups are amazing resources, with their membership size evidence of the usefulness to physicians. PMG participants write about how much medicine they learn, joking that it’s like CME (continuing medical education required for license maintenance). Could we create tools like this that are HIPAA compliant, enable documentation, and still have the utility?

The answer so far is “maybe.” Doximity is a social network for doctors, and it enjoyed a bit of a revitalization in the early days of the COVID-19 pandemic due to its video conferencing feature. Zingle, TigerText, DocHalo, and other companies have been developed around the idea of HIPAA-compliant texting. But none have the user base that WhatsApp has in Israel or PMG has on Facebook. For these tools, doctor doesn’t have to go somewhere special to ask the question—access to the tool is built in to their daily routine. When one creates a new platform, the convenience decreases and the likelihood of broad uptake is lower.

Some solutions to using everyday tools may be technological—perhaps there could be a way to authenticate on Facebook as a physician, or to submit WhatsApp messages into EHR documentation. But the greater barrier is the professional orcultural norm. How do doctors expect to communicate and what are they willing to share—with other doctors they know or don’t know—as part of a community? Are they willing to be contacted outside of the hospital systems? Are they willing to give considered but rapid advice, and to physicians not in their own medical network?

The Israeli doctors rated WhatsApp “as a highly effective tool for improving healthcare delivery.” But of course, it’s not just the tool but how it’s used. How could these tools be used? How should they be used? These are the questions we still face as we consider integrating better communication technologies into medicine.

## Data Availability

Not applicable.
